# Future challenges for UK regulation of brain organoid research

**DOI:** 10.1093/medlaw/fwae047

**Published:** 2024-12-26

**Authors:** Emily Jackson

**Affiliations:** Law School, London School of Economics and Political Science, London WC2A 2AE, United Kingdom

**Keywords:** brain organoid, cerebral organoid, human cerebral organoid, human tissue research, regulation, stem cells

## Abstract

One of this century’s most dramatic scientific developments is the reprogramming of stem cells in order to create organoids, that is, self-organizing 3D models that mimic the structure and function of human organs. This article considers whether brain organoids in particular might raise any new questions for law, now or in the near future. If complex human brain organoids were to become capable of consciousness or sentience, the current regulation of human tissue research, which protects the interests of tissue donors, might need to be supplemented in order to protect the interests of the tissue itself. Human brain organoids can also be implanted into animal hosts, and if this were to result in animals with significantly enhanced cognitive abilities, additional protective measures might become necessary.

## I. INTRODUCTION

Organoids are 3D models of organs and tissues created from either human embryonic stem (hES) cells or, more commonly, induced pluripotent stem (iPS) cells. Typically derived from skin cells, iPS cells have been reprogrammed into an ‘embryonic-like pluripotent state’,^1^ which means that they can become all of the different tissues and cells of the human body. In 1999, scientists reported having made cardiomyocytes (tiny muscle cells responsible for the contraction of the heart) from bone marrow stem cells.^2^ Now, it is possible to create ‘self-organising 3D structures, generated *in vitro* from stem cells, that resemble *in vivo* organs in terms of their structure and function’.^3^ Chosen as *Nature Methods*’ ‘method of the year’ in 2017,^4^ and contributing to what Charis Thompson has called the ‘in vitroization’ of biomedical research,^5^ organoids enable scientists to have ‘unprecedented access to [living] human tissue in the dish’.^6^

Different types of organoids give rise to different ethical and legal dilemmas. For example, *in vitro* gametogenesis (ie, the creation of sperm and eggs from iPS cells) has the potential to replace gamete donation with gamete *manufacture*^7^ for people who cannot reproduce with their own gametes.^8^ Questions are currently being asked about whether stem cell-based embryo models should be subject to any of the restrictions that apply to research on embryos.^9^ My focus in this article is instead on brain (or neural or human cerebral) organoids, and whether they might raise any new questions for the law, now or in the relatively near future.

Currently, in the UK, while research on human embryos is strictly regulated, there is comparatively little control over what scientists can do with other sorts of human tissue, provided that the original tissue donor gave informed consent. Researchers are expected to specify what they will do in the event of ‘incidental findings’, that is, if they find out information relevant to the tissue donor’s health.^10^ They must also ensure that there are effective data protection measures in place and that proper records are kept, so that materials are traceable.^11^ In short, the regulation of human tissue research is mainly directed towards protecting the interests of the tissue donor.

The creation of complex human brain organoids gives rise to a new possibility: that human tissue *in vitro* might become capable of rudimentary consciousness or sentience. If a brain organoid could develop awareness or the capacity for positive or negative experiences, then—for the first time—it may be necessary for the law to concern itself not only with the interests of the donor, but also with the interests of the tissue itself. There is no precedent for the regulation of research on *in vitro* human tissue that could experience harm or suffering (though it is worth noting that similar questions are being asked about the capacities of artificial intelligence^12^).

Additional ethical issues arise from the implantation of human brain organoids into animal hosts in order to stimulate growth and development. While transplanting human tissue into animals is not new, the creation of chimaeras^13^ with human brain tissue may be more unsettling than the incorporation of human DNA into an animal’s liver or kidney. Because it involves ‘protected animals’, the creation of animal/human chimaeras is already subject to regulation in the same way as other research on animals. If evidence emerged that the transplantation of human brain organoids significantly enhanced animals’ cognitive abilities, additional protective measures might become necessary.

This article begins with a brief description of brain organoid research, before turning to some of the ethical questions that arise from the creation of living human brain tissue. It then sets out how this sort of research is currently regulated in the UK and considers models for future regulation. Ultimately, it is important to consider what sort of limits might need to be placed on brain organoid research in order to reap its scientific and clinical benefits while simultaneously preserving public trust and confidence.

## II. BRAIN ORGANOIDS

### A. What are brain organoids?

In 2008, Mototsugu Eiraku and others first reported using hES cells to model the cerebral cortex.^14^ Five years later, Madeline A Lancaster and others ‘established a novel approach to studying human neurodevelopmental processes through *in vitro* culture of cerebral organoids from human pluripotent stem cells’, which could be used ‘to model aspects of human neurodevelopment and neurological disease and … provide novel insight into pathogenesis of these disorders’.^15^

The lack of effective treatments for many neurological and psychiatric disorders is in part due ‘to the difficulty of conducting research on an organ containing nearly 100 billion neurons interconnected by trillions of synaptic connections in intricate circuits that can hold vast amounts of information’.^16^ Until recently, research into human brain disorders had to rely upon brain tissue donated post-mortem, brain imaging carried out on living volunteers, or animal models, each of which has obvious limitations.^17^ Self-organizing 3D brain surrogates generated from iPS cells ‘recapitulate aspects of the developing human brain in vitro’^18^ and enable scientists to carry out research on live human brain tissue.^19^

In 1981, as a thought experiment in order to question how we could ever be sure that our experiences of the world are real and not simulations, Hilary Putnam posited a ‘brain in a vat’:

A human being … has been subjected to an operation by an evil scientist. The person’s brain … has been removed from the body and placed in a vat of nutrients which keeps the brain alive. The nerve endings have been connected to a super-scientific computer which causes the person whose brain it is to have the illusion that everything is perfectly normal.^20^

Brain organoids created from iPS cells are not fully functioning brains, like that imagined by Putnam. As J Lomax Boyd and Nethanel Lipshitz explain ‘[t]ypically, organoids are brain parts, but the possibility of a whole-brain-like organoid or, more likely, a whole-brain-like assembloid cannot be ruled out’.^21^ Lack of vascularization (which as we see later might be solved by implantation in an animal) will prevent brain organoids from growing larger than about 4 mm in diameter,^22^ or the size of a peppercorn.^23^ It should, however, be noted that some animals with tiny brains, like bees, appear to be capable of learning solutions to puzzles and experiencing emotions.^24^

A further obstacle to consciousness may be the lack of a functioning brainstem, and it is not yet known whether it would be possible to create an organoid with a brainstem.^25^ In addition, organoids do not have any of the sensory inputs ‘that may be necessary for the iterative learning and conditioning that cultivate cognitive processes’.^26^ Most experiential states—such as hunger and pain—result from the interaction between our brains and our bodies, rather than taking place in the brain alone.^27^

Leaving aside whether there could be ‘islands of awareness’ in a disconnected brain,^28^ interaction can be simulated by connecting brain organoids to other cells, such as retinal cells to generate a response to light, and muscle tissue to model muscle contraction. In more complex ‘assembloids’, brain organoids are connected to other organoids in order to ‘provide information about the communication between the brain and other organs, such as the gut–brain relationship’.^29^ Brain organoids can also be connected to ‘controllable robotic bodies’,^30^ and what has been described as organoid intelligence involves harnessing organoid cell cultures with ‘new forms of biocomputing’.^31^ Biological computing systems ‘could be faster, more efficient, and more powerful than silicon-based computing and artificial intelligence’,^32^ differing ‘significantly in the speed of computation and the energy required’.^33^

Concerns about brain organoids’ potential to develop the capacity for consciousness or sentience were boosted by research that demonstrated that ‘six-month-old human cortical brain organoids display electroencephalogram (EEG) activity patterns that resemble the electrical activity seen in 25–39 week-old premature infants’.^34^ That does not mean that brain organoids are the same as the brains of premature babies, however.^35^ Hyun and others caution that, currently, ‘too little is known about how babies’ brains are actually wired to make solid comparisons between organoids and naturally developing human brains in utero and neonatally’.^36^ Nevertheless, this uncertainty makes it difficult to be sure that brain organoids could not become capable of consciousness. Indeed, if we could rely on gaps in our understanding as a justification for treating brain organoids, which resemble babies’ brains, as if they were non-sentient, there would be a perverse incentive against trying to learn more about the capacities of both premature babies and brain organoids.

One of the most striking illustrations of the potential of brain organoids was the creation, by Brett Kagan and others, of *Dishbrain*: ‘a system that harnesses the inherent adaptive computation of neurons in a structured environment’^37^ and was taught to play the simple video game *Pong.* As Kagan and others explained,

Using this *DishBrain* system, we have demonstrated that a single layer of *in vitro* cortical neurons can self-organize activity to display intelligent and sentient behavior when embodied in a simulated game-world… These findings provide a promising demonstration of an SBI [synthetic biological intelligence] system that learns over time in a systematic manner directed by input.^38^

### B. Potential clinical use?

Brain organoids have already been used to develop a treatment for maternal transmission of the Zika virus. After identifying high rates of microcephaly, a severe brain disorder, among babies whose mothers were infected with the Zika virus, scientists exposed a brain organoid to the virus, which they found ‘was killing some of the intermediated neural progenitor cells, causing brain malformations similar to those that were manifested in babies’.^39^ This effect had not been seen in mice, and the researchers explained that the advantage to using ‘brain organoids that mimicked human neurodevelopment so well’ was that, within two years, they were ‘able to find a drug that would protect the infected mother from having a baby also infected with the Zika virus’.^40^

In the future, precision or personalized medicine might involve testing the efficacy of medication on an organoid created from the patient’s skin cells.^41^ Deriving patient-specific organoids before prescribing medication would likely be expensive,^42^ however, at least in the short term, so it is unlikely to wholly eliminate the cheaper ‘trial and error’ approach to medication.

More invasive, risky, and challenging would be the use of brain organoids in transplantation in order to replace a damaged part of a brain, perhaps following traumatic brain injury. Stem cell-derived retinal tissue has been used successfully in transplants,^43^ but brain organoid transplantation would be much more challenging. If the transplanted brain tissue affected ‘thoughts and behavior in an unintended or unpredictable way’, removing it might not be straightforward.^44^ It is, however, worth noting that, if it were to become safe enough, because the organoid is a clone of the person whose cells were used to create the iPS cells, recipients would not be dependent upon lifelong immunosuppression.^45^

The possibility of restoring functionality to a person’s brain also raises the question of whether this ‘could potentially undermine the diagnosis of brain death’.^46^ Even if we are sure that restoring a few neurons will not be able to re-establish brain function in someone who has died, sensationalist media representations of brain organoid transplantation could undermine public confidence in cadaveric organ donation (in the same way as an inaccurate 1980 *Panorama* documentary, which questioned whether donors were ‘really dead’).^47^

## III. CONSCIOUSNESS AND SENTIENCE

### A. Difficulties in defining and measuring consciousness and sentience

The difficulty of deciding what to do about the prospect of rudimentary consciousness and sentience in a brain organoid is exacerbated by disagreement over what consciousness and sentience mean, and how to measure them.^48^ Consciousness and sentience are not necessarily the same thing, although the terms are often used interchangeably. Dictionary definitions of consciousness refer to ‘being aware of and responsive to one’s surroundings’, while sentience indicates ‘a capacity to experience sensation’. Even this is contentious, however. There are those who claim that sentience exists in entities that merely respond to external stimuli, such that plants should be considered ‘sentient’,^49^ while others suggest that, to be sentient, an entity must be able to have positive and negative experiences, or ‘a life of her own, a life which matters *to her*’ (emphasis in original).^50^

Hank Greely has described consciousness as ‘one of those slippery words that, at least in English, gets used in many different ways with many different meanings and many different synonyms’.^51^ If there is ‘a lack of consensus about how to conceptualize consciousness’ and ‘[d]ifferences in how the concept of consciousness is understood’, it will be difficult to agree on an appropriate methodology with which to measure it.^52^ If, as Gardar Arnason and others suggest, ‘we don’t even agree on what we are looking for, even in healthy human adults’, how could we identify consciousness ‘in novel beings in the laboratory?’^53^

It is also possible that consciousness in brain organoids may emerge ‘in a different manner than in humans’ and ‘be associated with different biological underpinnings than in humans’,^54^ thus creating the risk that we may wrongly detect either the potential for consciousness or its absence. To make matters worse, Jacob Jeziorski and others warn that our ‘ability to reliably and uncontroversially detect cognitive and experiential capacities of organoids is likely to lag behind our ability to construct ever more complex neural organoids’.^55^

If we are looking for evidence of an entity’s mental state, this is ‘necessarily hidden from direct observation’.^56^ In animals, it is possible to rely on markers of sentience, such as self-administering analgesics after an injury, but this will not work in an organoid. Although it is hard to judge consciousness and sentience in an entity that cannot communicate or exhibit behaviour in response to sensory inputs, it is possible to measure electrical activity. Whether this is helpful is also a matter of dispute, however. Rachel Ankeny and Ernst Wolvetang suggest that consciousness is strongly correlated with irregular low-amplitude EEG activity,^57^ while Nita Farahany and others maintain that ‘the signals for consciousness or unconsciousness detected in a living adult–using electroencephalography electrodes, for example–don’t necessarily translate to infants, animals or experimental brain surrogates’.^58^

Despite these evidential difficulties, there is widespread, but not universal, agreement that the chance that the brain organoids created to date have any sort of awareness is currently ‘extremely low’.^59^ At the 2019 Society for Neuroscience meeting, some American neuroscientists claimed that brain organoid models might already have achieved some sort of consciousness and called for a complete moratorium on brain organoid research until effective screening mechanisms had been put in place.^60^ Taking the opposite view, Masanori Kataoka and others maintain that:

Even the most primitive consciousness would require a fairly complex and extensive neural architecture, which brain organoids cannot recapitulate. Therefore, issues related to the consciousness of organoids are highly speculative and not of pressing importance.^61^

Between these two extremes, the more common view is that the impossibility of being certain that brain organoids are permanently incapable of developing consciousness or sentience is itself ethically significant.^62^ While most scientists are currently confident that, to paraphrase Thomas Nagel, ‘there is not something it is like to be an organoid’,^63^ this is not the same as being sure that this is impossible. Furthermore, the science is moving only in one direction: towards more complex and interconnected organoids.^64^

### B. A precautionary approach?

The development of increasingly complex brain organoids, combined with uncertainty about their capacity for consciousness or sentience, has led some to advocate a precautionary approach.^65^ Although there are multiple versions of the ‘precautionary principle’,^66^ its origins lie in environmental policy, on the grounds that ‘where there are threats of serious or irreversible damage, lack of full scientific certainty shall not be used as a reason for postponing cost-effective measures to prevent environmental degradation’.^67^ Or, to put it another way, if human action might be causing a very serious bad outcome, it is sensible to set a low evidential bar when deciding whether to take steps to try to avoid that outcome.^68^

Jonathan Birch has described how the precautionary principle might work in relation to potentially sentient animals:

In broad terms, the idea is clearly that we should not require absolute certainty that a species is sentient before affording it a degree of legal protection. Absolute certainty will never be attained (indeed, the ‘problem of other minds’ suggests it cannot even be attained with respect to human minds), and its absence is not a good reason to deny basic legal protections to potentially sentient animals.^69^

Applied to brain organoids, a precautionary approach would mean that if there is doubt over whether an organoid could be sentient or conscious, we should not wait for definitive evidence before we start to treat them as if they were capable of consciousness and sentience. Brain organoids do not have pain receptors, but if they were used to create neural states akin to pain in order to test a new analgesic, it might be important to try to work out if there is any risk that the organoid could actually experience pain, ‘as opposed to merely display[ing] neurological features similar to a human brain experiencing pain’.^70^

At first sight, adopting the precautionary principle in the face of uncertainty about organoids’ consciousness might look sensible and innocuous, but if an unwarranted presumption of consciousness halts research that could generate effective treatments for serious brain disorders, there will be patients who are harmed by an overly cautious approach.^71^ It does not necessarily make sense to stop research into a possible cure for Alzheimer’s disease in order to protect the unknown interests of ‘a very simple life form that we would usually be willing to sacrifice in the face of the interests of a human being’.^72^

Tomasz Żuradzki suggests that there are two possible errors we could make in deciding whether to carry out research on entities where 100 per cent certainty about sentience is not possible: the first is to permit and conduct research on surrogates that in fact have a higher moral status, and the second is to forbid research when surrogates in fact do not have this higher moral status.^73^ To adopt a precautionary approach, according to Żuradzki, is to assume that avoiding the first error is more important than avoiding the second, when this is a claim that would itself require further justification.^74^

Żuradzki may be right to caution against invoking the precautionary principle to justify a complete ban on brain organoid research, but this assumes a strongly precautionary approach, where elimination is the only reasonable response to an unknown risk. It would be possible to adopt a more moderate version of the precautionary principle by seeking to minimize rather than eliminate any suffering.^75^ Even if we do not have definitive evidence of consciousness, we could adopt similar restrictions to those that apply to research on sentient animals, such as the 3Rs approach (Replacement, Reduction, and Refinement),^76^ supplemented by Tom Beauchamp and David DeGrazia’s principles of animal research: the principle of sufficient value to justify harm; the principle of no unnecessary harm; and a principle of basic needs.^77^ Applied to organoids, this would mean that models known to be non-sentient should be used wherever possible, the potential benefits of the research should justify any potential harms, and attempts should be made to reduce any risk of suffering.^78^

### C. A preference for ‘imperfect’ models?

It has been suggested that one way to address the ethical dilemmas associated with brain organoids might be to permit only the creation of very simple brain organoids, which we could be confident are incapable of consciousness or sentience.^79^ Using stress research as an example, Katherine Bassil and Dorethee Horstkötter suggest that there may be a need to avoid a situation in which the models become ‘“too good” and might themselves be harmed in the process of stress research’.^80^ Hank Greely sums up the conundrum:

When we avoid unethical research by making living models of human brains, we may make our models so good that they themselves deserve some of the kinds of ethical and legal respect that have hindered brain research in human beings. If it looks like a human brain and acts like a human brain, at what point do we have to treat it like a human brain—or a human being?^81^

Of course, if an incomplete model can serve the same purpose as a complete one, it will often simply be easier to use an incomplete model. It is also possible to envisage future regulation mandating the use of the least complex model necessary to carry out the research. But if a more complete model would be more useful for a particular research project, it may be preferable to allow research within strict regulatory limits, rather than mandating the creation of less useful models as a way to sidestep the ethical dilemmas to which more complex models give rise.^82^

## IV. MORAL STATUS OF A BRAIN ORGANOID

### A. What is the moral status of a brain organoid?

The term ‘moral status’ is often used as a shorthand for ‘the idea that some beings matter morally in their own right, and that their welfare, interests and choices carry non-negligible moral weight’.^83^ In addition to disagreement over whether this is helpful,^84^ there is also some debate over whether sentience is the only thing that matters,^85^ or whether it is necessary to balance the potential for suffering with other considerations, such as the capacity to form deep emotional connections with others.

Prioritizing suffering, Jeremy Bentham asked nearly 150 years ago, ‘the question is not, Can they reason? nor, Can they talk? but, Can they suffer?’,^86^ or, as Peter Singer put it more bluntly, ‘pain is pain’.^87^ At the same time, Singer himself has said that it does not follow from the ‘equal consideration of interests’ that killing a chicken is as seriously wrong as killing a human being, in part because the human has ‘differing capacities’, as well as people who love and care for her, who will suffer as a result of her death.^88^ In a similar vein, Jeff McMahan has said that killing a cow is less seriously wrong than killing a person, even though ‘it is less intuitive … to suppose that the physical suffering of a cow in itself matters less than the equivalent suffering of a person’.^89^

Although not everyone would accept this, it has been suggested that a creature’s complexity affects what counts as a harm for it.^90^ Adopting a capacities approach, Martha Nussbaum suggests that what matters is what it means for that species to live a flourishing life, so that it ‘would make no sense to complain that a worm is being deprived of autonomy, or a rabbit of the right to vote’.^91^ Even if it is true that ‘pain is pain’, humans experience psychological suffering, perhaps as a result of imagining and fearing their own death, in a way that *most* animals cannot. Importantly, however, evidence that some animals do have a concept of death and engage in elaborate mourning rituals should lead us to take seriously the idea that there might be other creatures who suffer as a result of fearing or witnessing death.^92^

It is often said that ordinarily adult humans have full moral status, while inanimate objects have no moral status, and in between these two extremes, there are entities with intermediate and/or uncertain moral status, such as non-human animals, foetuses, and perhaps also now brain organoids.^93^ Adult humans with capacity are not merely sentient; they also have a sophisticated concept of self over time, including a sense of their own future and their past, and they are capable of exercising autonomy and behaving reciprocally.^94^ Entities that may have some of these characteristics, but not all of them, might fall into the ‘intermediate’ category.

The familiar problem with this view is that it is normally assumed that all human persons—including newborn babies and people with cognitive impairments—have full moral status, even though they do not possess all of these characteristics, or do so to a lesser extent than some non-human animals.^95^ In what Joshua Shepherd has described as a ‘members first’ approach to moral status,^96^ it is also striking that the characteristics said to ground full moral status are typically human, so that the moral value of non-human animals tends to be judged according to their similarities to us.^97^

In relation to brain organoids, it seems clear that they are very far from achieving full moral status. Given how little we know about their capacities, it might also be premature to say that they have intermediate moral status; it may be more accurate to admit that their moral status is uncertain. As we have seen, most people consider that brain organoids are not capable of having pleasant or unpleasant experiences, but if this were to change, the organoid would acquire a degree of moral status, meaning that it matters for its own sake, and its interests would become relevant to the question of how it would be reasonable to treat it.

If the brain organoid is implanted into a rodent, pig, or non-human primate, the resulting chimaera would have an intermediate moral status, because—as a sentient creature—we know that it can be harmed. The difficult question is whether the fact that it is a humanized animal means that its interests matter more than they did before the transplant of the human organoid (a question I return to below). Moreover, by blurring species’ boundaries, chimaeras may pose an additional challenge to species-specific accounts of moral status.

### B. Are organoids things, persons, or hybrids?

Organoids are not persons and may be more accurately described as things or objects, but at the same time, they are alive and human, making them a rather special type of object or hybrid entity.^98^ As Amy Hinterberger and Sara Bea put it, ‘organoids are more than cells but less than organisms’.^99^ The public outcry following the retained organs scandals at Alder Hey and Bristol hospitals, in which it was discovered that human organs had been stored without consent,^100^ should lead us to take seriously these objects’ hybrid status as ‘human things’. In addition to concerns about possible sentience,^101^ measures would need to be in place to ensure ‘responsible stewardship’,^102^ such as not using, storing, or disposing of the organoid in ways that contravene the donor’s original consent.^103^

Brain organoids are usually created using iPS cell lines obtained from biobanks, when the tissue donors have given generic or ‘broad consent’, though some have argued that—for controversial uses, including creating a potentially conscious entity—broad consent might be insufficient and retrospective specific consent should be sought.^104^ It will also be important to have robust privacy and data protection measures in place: because the organoid is genetically identical to the donor, anonymization is impossible,^105^ and tests on the organoid might reveal sensitive medical information about a donor’s neurological disorder or mental state.

There is insufficient space here to do justice to debates over whether there are, or should be, property rights in human biomaterials.^106^ It is, however, generally accepted that, while property rights in the human body are exceptional,^107^ the application of human skill can convert separated tissue into property,^108^ and hence organoids could be owned and transferred for value. Ownership of an organoid would currently vest in the researchers or the institution in which the research is being carried out. The patenting of organoid technologies is already gathering pace, with hundreds of new patents each year.^109^ At present, organoids seem self-evidently patentable, since they fulfil the general prerequisites of invention, novelty, inventive step, and susceptibility of industrial application. In the future, if there is evidence of the capacity for consciousness or sentience, questions may be raised about whether brain organoids might become unpatentable on the grounds that they would be ‘inventions the commercial exploitation of which would be contrary to *ordre public* or morality’.^110^

Intellectual property rights are, of course, a double-edged sword. On the one hand, they can drive innovation and accelerate progress. On the other, they could make any resulting clinical interventions prohibitively expensive, and therefore accentuate and widen health inequalities. The commercialization of organoid technology also raises the question of whether the person who donated the skin cell might have any interest in the profits derived from organoids created from their tissue. In practice, one person’s skin cell donation will almost certainly not have particular commercial value (akin to the HeLa cell line derived from Henrietta Lacks’ cervical tumour in the 1950s).^111^ More commonly, research will proceed slowly and incrementally, with organoids created from multiple donors being used at the same time.

Indeed, commercialization is unlikely to be the most pressing issue raised by the repeated use of particular donors’ cell lines in organoid research. In practice, a more important concern may be ‘to ensure that the cells and tissues used to create organoids are representative of diverse populations’, so that ‘the research is relevant and applicable to a wider range of people’.^112^ It might also be necessary to take steps to promote trust among marginalized communities in order to encourage them to become tissue donors for stem cell research.^113^

## V. BRAIN ORGANOID RESEARCH AND ANIMALS

### A. Chimaeras

Although not being connected to a blood supply limits a brain organoid’s development, this problem can be solved by implanting it in an animal host. In 2018, Abed AlFatah Mansour and others reported the successful transplantation of human brain organoids into adult mice, and

demonstrate[d] that human brain organoids are amenable to transplantation in the rodent brain, showing integration, viability, long-term survival, vascularization, functional neuronal activity, and synaptic connectivity of the grafted organoid and the host.^114^

While transplantation enables organ growth that is not possible *in vitro*, it is only likely to result in a brain with a tiny fraction (no more than 0.5 per cent) of the number of cells in the brain of an adult human.^115^ Furthermore, at the moment, transplanting a human brain organoid into an animal might even be ‘more likely to worsen brain function than improve it’.^116^

Nevertheless, it is clearly possible that, in the future, animals implanted with human brain tissue might develop enhanced cognitive capacities. If this were to happen, the research might have to be subject to additional restrictions, in the same way as research on non-human primates. For example, destroying the chimaera after the experiment is over might be unacceptable,^117^ and enhanced chimaeras might have to be ‘retired to colonies such as chimpanzee sanctuaries’.^118^ In order to preserve public trust, tissue donors may have to be informed that their cell lines could be used to create chimaeras, so that their broad consent covers this eventuality, and anyone who objected would have the option of opting out.

The ‘humanisation’ of animal brains could result in beings with a ‘morally ambiguous status’,^119^ particularly if non-human primates were to be used. There have been human brain organoid experiments involving macaques, but the transplantation was limited to the motor cortex, ‘‘[i]n order not to affect the higher brain functions of the monkeys’.^120^ Non-human primates’ ‘evolutionary proximity’ to humans might ‘constitute a scientific advantage’ for human brain organoid transplantation, and make it easier to recognize ‘symptoms of psychiatric disorders, including sadness, decreased interest in activities, disordered sleep, and social isolation’.^121^ At the same time, they could ‘evoke the specter of more human-like intermediate animals, heightening concerns about the blurring of boundaries between species’.^122^

There are those who believe that species boundaries have moral significance.^123^ Jason Scott Robert and Françoise Baylis, for example, argue that even though:

Scientifically, there might be no such thing as fixed species identities or boundaries. Morally, however, we rely on the notion of fixed species identities and boundaries in the way we live our lives and treat other creatures.^124^

According to Robert and Baylis, crossing species boundaries involves violating a valuable taboo against mixing humans and animals, and ‘represent[s] a metaphysical threat to our self-image’, with the potential to ‘introduce inexorable moral confusion in our existing relationships with non-human animals and in our future relationships with parthuman hybrids and chimeras’.^125^ Similarly, it has been argued that the creation of chimaeras would ‘denigrate human dignity’ by:

giving nonhumans some of the physical components necessary for development of the capacities associated with human dignity, and encasing these components in a nonhuman body where they would either not be able to function at all or function to a highly diminished degree.^126^

Of course, for those whose religious faith leads them to believe that man was created in God’s image, treating human beings as entirely separate and distinct from other animals may make sense. The scientific and biological reality, however, is that humans evolved from an ape ancestor, and, as Benjamin Capps explains, ‘scientific methods have yet to prove any benchmark moral properties that are distinctly human (some humans lack corresponding capacities) or that are entirely absent in animals’, nor are humans ‘singularly conscious beings, or uniquely communicative’.^127^

The mixing of animal and human material already happens in various ways, such as the use of pig heart valves in human transplantation and the creation of transgenic animals for research purposes, as well as potential sources of organs for transplant. Unless all such research is deemed unethical, the important question will be whether it is possible to identify a boundary between permissible and impermissible research involving the transplantation of human brain organoids into animal hosts. For example, could we point to a level of cognitive capacity or a marker of distress that would render unethical the creation of a chimaera that reaches that capacity or displayes that marker? Should there be a moratorium on the transplantation into non-human primates of brain organoids that might be capable of affecting their ‘higher brain functions’?^128^

### B. Relationship between brain organoids and research on animals

Even if, as Insoo Hyun and others have claimed, the prospect of conscious and sentient brain organoids is ‘extremely remote’,^129^ there has nevertheless been considerable agonizing over how they should be treated. In contrast, we know that sentient animals, capable of experiencing pain and fear, are routinely used in research and in the production of meat.^130^ If the goal is to reduce suffering in animals, organoids, including brain organoids, could be used in order to replace or reduce the use of animals in research, at the same time as allowing greater experimental flexibility at a lower cost.^131^ Indeed, organoids will often be better models than animals for drug screening, given that they are human and can even be tailored to be patient-specific.^132^

It would, however, be too hasty to suggest that organoids could entirely *replace* research on animals. As Hinterberger and Bea point out, while ‘some studies can be run on organoids alone, bioscience journals commonly require in vivo proof of study claims’.^133^ Moreover, the implantation of human organoids in animal models may actually increase the number of animals used in research.^134^ Organoids could therefore contribute to the 3Rs approach by replacing some animals, while at the same time disrupting the 3Rs approach by creating a whole new field of research reliant on the use of animal models.

Could it furthermore be argued that if research on animals is allowed when we *know* that they are sentient,^135^ brain organoids could also be used in research *despite* any potential they might have for sentience (while perhaps also applying a 3Rs approach)?^136^ Or does the fact that a brain organoid is human mean that research should be stopped before there is the slightest chance of sentience (in the same way as we would not allow researchers to dissect the brain of a living human being)?

## VI. REGULATING BRAIN ORGANOID RESEARCH

Thus far, I hope I have established that brain organoid research has the potential to raise novel issues for regulation. Scientists are currently creating and using brain organoids in research, subject only to the restrictions that cover other uses of banked stem cell lines, such as ensuring that any uses are covered by the tissue donor’s consent and protecting the donor’s identity. The overarching question we need to ask ourselves is whether there are any circumstances in which research, even if it adequately protect the tissue donor’s consent and privacy interests, could nevertheless become unethical. If there are, a new regulatory response may be required.

Before considering what this might look like, I will first describe the law which currently applies to brain organoid research in the UK, as well as the ‘soft law’ or guidance set out in the current (but soon to be revised) guidelines from the International Society for Stem Cell Research (ISSCR). Next, I will consider whether two existing models for regulation—the laws that apply to embryo research and to research on animals—might serve as a useful starting point for a new regulatory framework. Given that one of the purposes of regulation is to provide reassurance to the public, so that, as Mary Warnock put it, ‘they can be certain that no nameless horrors are going on, hidden away in laboratories’,^137^ further public engagement will be necessary, in order to find out more about people’s attitudes towards brain organoid research. Finally, I will propose a synthesis of principles from three existing bodies of law in order to offer adequate protection not only to tissue donors but also to the wider public, to animal/human chimaeras and, in time perhaps, to the brain organoid itself.

### A. Regulation of human tissue research in the UK

In the UK, the Human Tissue Act 2004, administered by the Human Tissue Authority (HTA), applies to the removal, storage, use, and disposal of human tissue. The central organizing principle of the Act is that the use of ‘relevant material’ for ‘scheduled purposes’, including ‘research in connection with disorders, or the functioning, of the human body’,^138^ requires ‘appropriate consent’.^139^ Consent to the use of human tissue in research must be a ‘positive act’,^140^ rather than being presumed or ‘deemed’, as can now be the case for cadaveric organ donation.^141^ In relation to human tissue research, if the donor is alive, then ‘appropriate consent’ means ‘his consent’.^142^

Consent is always required for the removal of tissue from a living person, unless they lack capacity and the removal is judged to be in their best interests (which will seldom be the case for tissue donation to stem cell research).^143^ Consent can be specific to a particular project or generic to cover a range of future uses. Tissue donors for stem cell research will usually be asked to give ‘broad consent’, so that cell lines derived from their cells will be available for use by unknown future researchers. In seeking broad consent, it is not necessary to define all possible future uses, but rather to ‘provide information to participants to help them understand the scope of future research use and what this might mean for them’.^144^

While the removal of tissue always requires consent (or a best interests decision), consent is not always necessary for the storage and use of human tissue under the Human Tissue Act 2004. For example, consent is not required for the use of tissue in research if it has already been taken from a living person (which would have required consent), but the researcher is unable to identify the donor, and the project has received ethical approval from a Research Ethics Committee (REC).^145^

In practice, the HTA’s role in the regulation of organoid research is limited. While the HTA ‘licenses organizations for removal and storage for research in England, Wales and Northern Ireland’,^146^ it does not ‘license the “use” of tissue for research or approve individual research projects or clinical trials’.^147^ If a research project has been approved by (or approval is pending from) a recognized REC,^148^ or has received samples from a REC-approved tissue bank, it does not require an HTA storage licence. In addition, because they were not removed directly from a human body, cell lines, cells that have divided in culture, and embryonic stem cells are categorized as ‘not relevant material’ for the purposes of the Human Tissue Act 2004. Research using cell lines does not require REC approval, either under the Human Tissue Act or NHS research governance arrangements.

If there is an intention to transplant tissues and cells into patients, the HTA would have a role in regulating the ‘procurement, testing, processing, storage, distribution and import/export of tissues and cells, including cell lines, intended for human application’.^149^ Brain organoids are unlikely to be intended for human application for some time, however.

If hES cells are used to create the organoid, and if the hES cell is derived within the project, researchers would need to obtain a licence from the Human Fertilisation and Embryology Authority (HFEA) and comply with the provisions in the Human Fertilisation and Embryology Act 1990. This would be unusual, however, and researchers would be more likely to obtain hES cell lines from the UK Stem Cell Bank, where all UK researchers deriving hES cell lines are required, as a condition of their HFEA licence, to deposit a sample of their embryonic stem cell line. The UK Stem Cell Bank Steering Committee is responsible for ‘ensuring that donor consents, ethical approvals, licences and authorisations are in place for all stem cell lines deposited in the UK Stem Cell Bank, and for all projects receiving cell lines from it’. ^150^

In practice, most brain organoid research involves the use of iPS cells, once again obtained from biobanks. Operated by facilities within the European Union and the UK, the European Bank for induced pluripotent Stem Cells (EBiSC) is a centralized, not-for-profit iPSC bank that provides ‘access to scalable, cost-efficient and consistent, high-quality iPSC lines’ to researchers worldwide. The EBiSC reviews the informed consent given at the time of sample collection in order to ensure that donors were fully informed that their cells might be used for iPSC generation, distribution and use, and to make sure that any restrictions placed on how lines could be used are respected. Scientists who wish to use lines from the EBiSC must complete an Access and Use Agreement, which specifies which uses are acceptable (research purposes) and which are not (identification of donors).^151^

### B. The ISSCR guidelines

#### 1. ISSCR guidelines on brain organoids

At the time of writing, the ISSCR is working on revisions to its 2021 guidelines, which treat brain organoids in the same way as other organoid research, and recommend that they should be ‘exempt from review by a specialized oversight process’.^152^ This was because, according to the ISSCR, there is ‘no biological evidence to suggest any issues of concern, such as consciousness or pain perception … that would warrant review through the specialized oversight process’.^153^ UK regulation of brain organoid research—which treats it in the same way as research on non-sentient human tissue—is therefore in line with the current ISSCR guidelines.^154^

If evidence emerged which gave rise to concerns about the organoid’s potential for consciousness or sentience, exemption from ‘specialised review’ is unlikely to continue to be appropriate. Were this to happen, scientists should not necessarily wait for new guidance or laws before they adapt their practices. According to Julian Koplin and John Massie, scientists should ‘consider the moral status of the entities [they] create’, rather than merely relying on where they fit in the regulatory landscape.^155^ The ISSCR acknowledges that there may be shifts in the evidence and recommends that ‘researchers should be aware of any ethical issues that may arise in the future as organoid models become more complex through long-term maturation or through the assembly of multiple organoids’.^156^ If organoids were to become sufficiently complex that their ISSCR categorization changed to being ‘reportable’ or subject to ‘full specialised review’, it would be necessary to identify a body in the UK that could be responsible for receiving reports or undertaking reviews.

#### 2. ISSCR guidelines on chimaeras

The ISSCR’s 2021 guidelines—fleshed out in a White Paper published by members of the ISSCR Task Force subcommittee—suggest that, at least for the next 5–10 years, the ‘transfer of human stem cells and their derivatives into postnatal hosts’ should be treated like other research on animals. This means it, too, is not subject to ‘full specialised review’, but should instead ‘continue to be reviewed by the usual animal research committees utilized by research institutions … [and] comply with the principles of the 3Rs (replacement, reduction, and refinement’.^157^

The White Paper does recommend some additional monitoring of chimeric animals:

Research with a known, intended, or well-grounded significant potential to create some aspect suggestive of human cognition, self-awareness, behavior or behavioral pathology, while not prohibited, should be subject to close scrutiny, taking care to ensure the humane protection of animal subjects.^158^

It also suggests that additional precautions may be needed in order to protect non-human primates and laboratory staff.^159^ But the ISSCR guidelines also advise against making ‘any statements implying human cognitive abilities, human consciousness or self-awareness, as well as phrases or graphical representations suggesting human-like cognitive abilities’, on the grounds that this ‘risks misleading the public and sowing doubts about the legitimate nature of such research’.^160^

As Julian Koplin has pointed out, in defending their permissive approach to chimeric animal research, the ISSCR does not just rely on evidence suggesting that there is unlikely to be substantial cognitive enhancement of chimeric animals in the near future. As well as claiming—controversially^161^—that it is only a ‘distinctly human form of self-consciousness’ that leads to an entity’s higher moral status, the ISSCR makes the ‘even more troubling argument’ that ‘moral status concerns can be nullified by raising chimeric animals under conditions that prevent them from developing the requisite cognitive abilities’.^162^ As Koplin points out, it would also be possible to raise human children under conditions that prevented them from developing ‘self‐consciousness or other morally relevant cognitive capacities’,^163^ but it is hard to imagine anyone claiming that doing so could facilitate the use of children’s bodies for research purposes.

### C. Existing models for regulation

The restrictions on human embryo research—set out in the Human Fertilisation and Embryology Act 1990, and resulting from the recommendations of the 1984 Warnock Report—are not intended to protect the interests of the individual embryo.^164^ Rather, their purpose was to safeguard what was described as the special moral status of human embryos, which required them to be treated ‘with respect’,^165^ or, as Mary Warnock since suggested would be more accurate, ‘non-frivolously’.^166^ Whether embryos acquire this special status because of their symbolic value,^167^ or because the disrespectful treatment of embryos might matter to sentient human persons,^168^ the law governing embryo research offers the most robust and restrictive model for the regulation of research on human tissue.

In order to carry out research on embryos, scientists must first obtain a licence from the HFEA. The HFEA’s Licence Committee can only issue a licence if it is satisfied that the research is necessary or desirable for one of the statutory purposes,^169^ and that the use of embryos is necessary (ie the research could not be done with banked hESC lines or with animal models).^170^ Donors must give specific consent for the use of their embryos in a research project,^171^ and it is a criminal offence to culture an embryo *in vitro* for more than 14 days or after the appearance of the primitive streak,^172^ whichever happens first.^173^

Most of these provisions would be excessively burdensome if applied to brain organoid research. For example, organoids’ potential to replace the use of sentient animals in research would be jeopardized if brain organoids could be used only if it was impossible to use animals instead. Nevertheless, the idea of a time limit or developmental marker beyond which embryos must not be allowed to develop might usefully be adapted in order to set a regulatory limit on brain organoid research, which could offer reassurance to the public as well as a clear boundary for scientists.^174^

If our primary concern is the organoid’s potential sentience, the laws that apply to research on animals may be a better fit, especially given their shift over the course of the 20th century from prohibiting cruelty (often explicitly on the grounds that ‘if such behavior was condoned in society, the perpetrators would be likely to advance to abuse of humans’^175^) to protecting animals’ interests *for their own sake*. In addition, of course, where brain organoid research involves the creation of chimaeras, it is already regulated in the same way as other research involving ‘protected animals’.

In the UK, it is unlawful to carry out research on protected animals without a licence from the Home Office. Section 3 of the Animals (Scientific Procedures) Act 1986 specifies that the laboratory, the individual researcher, and the project itself must all be separately approved. Researchers must provide evidence that they have been trained in the ethics of animal research, caring for animals, and identifying illness and distress. Laboratories must meet minimum standards of staffing, veterinary care, housing, lighting, ventilation, and temperature control. A Project Licence requires ethics committee approval, and the use of animals must be justified. The Secretary of State, whose role under the Animals (Scientific Procedures) Act 1986 is in practice delegated to the Animals in Science Regulation Unit and the Animals in Science Committee, must ‘assess the compliance of the programme of work with the principles of replacement, reduction and refinement’,^176^ and must:

carry out a harm-benefit analysis of the programme of work to assess whether the harm that would be caused to protected animals in terms of suffering, pain and distress is justified by the expected outcome, taking into account ethical considerations and the expected benefit to human beings, animals or the environment.^177^

The Animals (Scientific Procedures) Act 1986 thus has three main goals: justifying the use of sentient animals, minimizing their suffering, and prohibiting certain types of research (eg cosmetic testing and the use of Great Apes). Any regulation of the creation and use of potentially sentient brain organoids might similarly require their use to be justified, and any suffering to be minimized, while at the same time perhaps specifying some ‘red lines’, such as a requirement to stop research immediately if there is evidence of distress.

### D. Public engagement

Public engagement is increasingly acknowledged as an important and necessary step in the regulation of scientific innovation.^178^ The limited public engagement carried out to date suggests that there is broad support for research on organoids, while at the same time, people are concerned about excessive commercialization and its implications for health inequalities.^179^ As well as expressing a preference for public sector control and ethical oversight—in preference to peer review and self-regulation—there are anxieties about privacy, data protection, and informed consent.^180^ Because they raise ‘moral concerns’, some types of organoid, including gametes and brains, are commonly viewed as ‘morally distinct’.^181^ In the case of brain organoids, there are particular concerns about consciousness and sentience,^182^ and connecting brain organoids to other organoids, because ‘these activities edged closer to creating ‘‘life”’.^183^

Given that the brain is widely believed to be ‘the source or seat of (moral) reasoning, human behaviour, and cognitive functioning, and the “self”’,^184^ public fears about brain research may be particularly compelling and visceral. As Peter Reiner has put it:

It is not so much that we are not also our genes, our bodies, members of social groups, and so on, but rather that when we conceive of ourselves, when we think of who we are as beings interacting in the world, the *we* that we think of primarily resides in our brains (emphasis in original).^185^

As ‘more complex brain organoids are created, the possibility may arise that human individuals will be created’,^186^ with the fact that the organoid is a clone being especially unsettling.^187^ As Julian Koplin and others put it, ‘While creating a human brain organoid does not clone an entire person, it effectively clones the part that *matters*’ (emphasis in original).^188^ When describing how he felt about a brain organoid created from his skin cell, Philip Ball said:

I didn’t lie awake at night fretting over its welfare; this mass of tissue made from my skin didn’t take on the status of an individual. But I felt oddly fond of those cells … There was a curious intimacy involved, a sense of potential that wasn’t present initially in the tiny chunk of arm-flesh excised and placed in a test-tube.^189^

If the public shares Ball’s perception that a brain organoid’s ‘sense of potential’ makes it different in kind from the skin sample from which it was created, the status quo—in which regulation treats all human tissues (aside from embryos) in the same way—may be insufficient to secure public confidence in brain organoid research.^190^

Although not relevant to all research on brain organoids, where the intention is to seek cures for brain disorders, there is also evidence that the public is worried about perfectionism and the implications of eliminating neurological disorders that are not life-limiting, where those affected may consider themselves to be neurodiverse rather than ill.^191^ As Andrew Barnhart and Kris Dierickx explain, research on brain organoids tends to reinforce the medical model of disability, in which disability is caused by an abnormality or impairment.^192^ For some disorders, this is uncontroversial: few would argue that a cure for brain cancer would unreasonably decrease diversity. But for autism spectrum disorders in particular, the medical model is increasingly contested. It might therefore be important to include the voices of neurodiverse individuals in the planning of research, not because ‘co-creation’ will provide a definitive answer to the question of whether a condition is a disorder or a difference, but in order to promote broad public trust and confidence in brain organoid research.

Genuine public engagement involves dialogue with the public about what concerns them,^193^ rather than adopting the top-down ‘deficit’ model of public education.^194^ At the same time, it is worth acknowledging that there is currently little public awareness or understanding of organoid research, and so it will be important for scientists to offer clear and simple explanations of what this research involves and what it hopes to achieve.

While brain organoids could undoubtedly be useful in developing treatments for brain disorders like Parkinson’s and Alzheimer’s, it is important that explanations of brain organoid research do not ‘overpromise’^195^ and create ‘false hope’ that a cure for dementia is imminent.^196^ It is also important that scientists do not ‘underpromise’ about the potential of these new entities: the public might be reassured by statements that brain organoids are incapable of consciousness, but it is important to be honest that it is impossible to be certain that this could never be the case.^197^

Abigail Presley and others’ study of media coverage of brain organoids suggests that there has been increasing polarization, and articles are either unrealistically positive about imminent cures for brain disorders or alarmingly negative about self-aware ‘brains in vats’ and rodents with human brains.^198^ In addition to a sober evaluation of both the risks and benefits of brain organoid research, it is important not to lose sight of the fact that it also raises more mundane and familiar ethical issues, such as informed consent, confidentiality, and the need for oversight.

## VII. PRINCIPLES FOR FUTURE REGULATION

My claim in this article has been that existing regulation of human tissue research—with its focus on the interests of tissue donors—may need to be supplemented in a number of ways to address the additional ethical issues that might arise from brain organoid research.

First, it will continue to be necessary to protect donors’ interests and, in the context of brain organoids, to ensure that the consent of the original tissue donor is sufficient to cover whatever researchers subsequently do with cell lines derived from their donation. The traditional model of consent, where researchers obtain consent from donors for the use of tissue in *their* research project—when it is possible to be precise about exactly what is planned, so that the tissue donor can make an informed decision about whether to donate tissue for that particular use—has been superseded by biobanking, where the projects that seek to use biobanked tissues may not even exist when consent to donation was given.^199^ Of necessity, therefore, biobanks must rely upon tissue donors’ broad and unspecified consent.

In addition to the impossibility of obtaining specific informed consent, the Human Tissue Act 2004 treats cultured and banked stem cell lines as an exception to its normal requirements for consent to the use of human tissue. As a result, the creation of brain organoids from iPS cell lines is subject to hardly any regulation at all.

In the short term, therefore, decisions may have to be taken about whether the broad consent of tissue donors for iPSC research is sufficient to cover the creation of complex brain organoids and their implantation in animal hosts, or whether it might become necessary to recontact donors in order obtain specific consent. Recontacting donors is not straightforward; however, not only can it be difficult and expensive to track donors down, but also some donors may prefer not to be recontacted.^200^

Public engagement might be helpful in order to find out how the public, in general, and tissue donors, in particular, feel about cell lines derived from their tissue being used to create a brain organoid that might be implanted in an animal host, or that might become capable of rudimentary sentience. When obtaining broad consent in the future, it may be sensible to give donors some indication of these possible uses of cell lines derived from their donation so that they have the chance to object or reconsider their donation.

Secondly, borrowing from the regulation of embryo research and proposals for the regulation of stem cell-based embryo models,^201^ it might be helpful to set a time or developmental limit in order to reassure the public that brain organoids will not be developed until they exhibit ‘morally concerning features’.^202^ Once again, public engagement may be helpful in identifying features or characteristics of a brain organoid that could serve as a marker for when research must stop. Whether this is expressed as a time or developmental limit, or a level of electrical activity, or some combination of different limits, ruling out the indefinite development of brain organoids *in vitro* might help to promote public confidence.

Thirdly, the law which covers animal research may offer the most useful initial framework for accommodating novel issues that arise from the creation of animal/human chimaeras with humanized brains, and in order to address the possible sentience of the brain organoid itself. In the short term, the Animals in Science Regulation Unit and the Animals in Science Committee should already be keeping chimeric research involving human brain organoids under review and should alert the Secretary of State if there is evidence of enhancement, such that additional restrictions on the treatment of chimaeras become necessary, for example, by safeguarding the welfare of animals after the research is over. Ensuring that animals with enhanced cognitive abilities are treated appropriately may require veterinarians and researchers to acquire new expertise in the welfare of chimeric animals.

Jonathan Birch has suggested that it might be sensible for the Animals in Science Committee also to be charged with keeping research on potentially sentient brain organoids under review. Not only are there obvious similarities between research on sentient animals and research on potentially sentient organoids, but also charging one body with reviewing both types of research would enable them:

to see the trade-offs involved in the two kinds of research, to advise on cases that blur the boundaries between the two (because an organoid is implanted into an animal), and to advise replacing animals with organoids where appropriate.^203^

This suggestion has obvious merit, and it is clear that the Animals in Science Committee already has an important role in relation to the creation of chimaeras. Nevertheless, in addition to synergies between research on animals and brain organoids, there may also be several differences that will need to be taken into account.

First, an underlying premise of the rules governing animal research is that ‘the importance of the scientific question being researched on animals takes precedence over the welfare of the animals’.^204^ Compulsory adherence to the 3Rs approach may have led to a decline in the use of animals since the peak of 5.3 million in 1972,^205^ but it is still consistent with the use in 2022 of 2,761,204 animals in experimental procedures, 2,197 of which involved monkeys.^206^ In terms of refinement, while the majority of experiments on animals in 2022 did not cause suffering above the threshold for regulation (defined as ‘less than the level of pain, suffering, distress or lasting harm that is caused by inserting a hypodermic needle according to good veterinary practice’), 15.5 per cent of experiments caused moderate suffering and 1.5 per cent—that is 41,418 procedures—caused severe suffering. We would need to ask ourselves whether potentially sentient brain organoids should be subject to the same standard of review as whole animals, thus opening up the difficult question of whether there is something special about human sentience, even if it only exists in a dish, or whether ‘pain is pain’.

Secondly, the suffering of a sentient human brain organoid may be different from the harms experienced by animals used in research. We may understand what it means to meet the basic needs of a mouse, but what would this involve for a potentially sentient brain organoid, and will it vary depending upon which brain region is recapitulated?^207^ The detection of suffering is also likely to be more challenging in a brain organoid than it is in a sentient animal. Proxies for sentience may be required, and decisions will need to be made about whether research must cease immediately if there is any evidence of sentience, or whether it would be sufficient to take steps to reduce but not eliminate suffering.

Thirdly, responsibility for judging the humane treatment of animals in research is delegated to veterinarians, who have expertise in animal welfare. There is no equivalent body of professional expertise for brain organoids, so it will be necessary to put in place mechanisms and training for the evaluation and monitoring of organoids’ experiential interests. Fourthly, because brain organoids are created from cells taken from human donors, it will continue to be necessary to protect the tissue donor’s interests, and there is no equivalent consideration in the law that applies to animal experiments.

Finally, there would be obvious difficulties if a 3Rs approach were applied to two different entities simultaneously: at the moment, it may make sense to use organoids in order to replace sentient animals, but if we also had to strive to replace sentient human brain organoids with something else, such as animals, the 3Rs approach would become unhelpfully circular.

Given that there are both similarities and differences between animal research and research on potentially sentient brain organoids, it might therefore be sensible to delegate overall responsibility for monitoring developments in brain organoid research to a new specialist committee. Whether this should be confined to brain organoids or should extend to organoid research more generally is open to question. A (Brain) Organoid Research Oversight Committee could draw upon relevant knowledge and experience from the Animals in Science Regulation Unit and the Animals in Science Committee, but might also benefit from the expertise of other bodies, including the Medical Research Council, the UK Stem Cell Bank Steering Committee, the Human Research Authority, the HTA, and the HFEA, as well as ensuring that ‘lay’ and patient perspectives are represented.^208^ The role of this Committee would—for the time being—simply be to oversee developments in (brain) organoid research, with a remit to advise the Secretary of State for Health and Social Care if evidence emerges that necessitates changes in the way in which (brain) organoid research is regulated. While the rules that cover research on animals may offer a helpful starting point when devising rules that aim to justify, minimize, or prohibit suffering, in practice, there will have to be organoid-specific rules that address the practical difficulties in detecting, minimizing, and preventing harm to human tissue *in vitro*.

## VIII. CONCLUSION

In the UK, no regulatory body has decision-making authority over brain organoid research. Its reliance on banked cell lines takes it outside of the scope of the HTA, and while research involving transplanting human organoids into animal hosts will be subject to the Animals (Scientific Procedures) Act 1986, there is no entity responsible for oversight of other organoid research.

Indeed, because the Human Tissue Act 2004 has little application to research using banked stem cell lines, organoid research is in practice subject to *fewer* restrictions than research on tissues taken directly from a donor. Biobanking also makes it impossible for tissue donors to give specific informed consent to each of the future possible uses of stem cell lines derived from their donation. In 2004, it may have made sense to subject banked cell lines to less rigorous regulation than research on directly removed tissue samples, but as organoid technologies become more sophisticated, this comparative lack of regulation looks increasingly untenable.

As part of its strategic focus on the ‘mind and brain’, the Nuffield Council on Bioethics (NCOB) produced a policy briefing note in early 2024, which acknowledged that the research ‘is moving at pace, and it is difficult to predict when significant developments will take place’ and stressed that it was:

important, therefore, for policy makers to work with scientists, ethicists, and publics to ensure that the ethical and regulatory questions are fully explored, in order to ensure that appropriate guidance and regulations will be in place to facilitate innovation and address ethical considerations.^209^

At the time of writing, the NCOB has issued a call for evidence to inform further ethical guidance, seeking in particular views on ensuring that regulation of neural organoids ‘can be proportionate and future proofed’; what an appropriate informed consent process might look like, given ‘fast-paced developments and an unpredictable direction of research’; and what possible characteristics of organoids ‘may warrant special ethical consideration’.^210^

Within the next year, there are likely to be recommendations from the NCOB, as well as updated ISSCR guidelines, and at some point, the government may need to decide ‘how the law ought to respond to the ethical issues raised by organoid research’.^211^ Whether this will require a ‘full ethics led inquiry’,^212^ akin to the 1984 Warnock Report,^213^ or whether the government devises its own proposals, perhaps relying on the NCOB’s forthcoming recommendations, doing nothing runs the risk of the science overtaking the law.

There is also a need to consider whether any new legislation should regulate organoid technologies in general, or whether brain organoids raise sufficiently distinctive issues that they should be regulated in a different way from, say, placental or kidney organoids. What seems clear, however, is that we should not wait until a brain organoid exhibits signs of sentience before we start to consider whether the law that applies to stored blood samples is fit for purpose when researchers are instead creating and experimenting on live human brain tissue.

## Footnotes

1 Xiao-yang Zhao and others, ‘iPS Cells Produce Viable Mice Through Tetraploid Complementation’ (2009) 461 Nature 86.

2 Shinji Makino and others, ‘Cardiomyocytes can be Generated From Marrow Stromal Cells in Vitro’ (1999) 103 Journal of Clinical Investigation 697.

3 Julian J Koplin and Julian Savulescu, ‘Moral Limits of Brain Organoid Research’ (2019) 47 The Journal of Law, Medicine & Ethics 760.

4 ‘Method of the Year 2017: Organoids’ (2018) 15 Nature Methods 1. Embryo models, described as ‘models for modelling development’ were named as the 2023 Method of the Year ‘Methods for Modelling Development’ (2023) 20 Nature Methods 1831.

5 Charis Thompson, *Good Science: The Ethical Choreography of Stem Cell Research* (MIT Press 2013). See also, Sarah Franklin, ‘Developmental Landmarks and the Warnock Report: A Sociological Account of Biological Translation’ (2019) 61 Comparative Studies in Society and History 743.

6 Amy Hinterberger and Sara Bea, ‘How do Scientists Model Humanness? A Qualitative Study of Human Organoids in Biomedical Research’ (2023) 320 Social Science & Medicine 115676.

7 Although some US biotech companies believe that they are close to carrying out clinical trials involving in vitro gametegenesis (IVG) (see, eg <https://conception.bio/>), in the UK new primary legislation would be necessary before it would be lawful to use IVG in fertility treatment.

8 Lauren Notini, Christopher Gyngell, and Julian Savulescu, ‘Drawing the Line on In vitro Gametogenesis’ (2020) 34 Bioethics 123; Timothy F Murphy, ‘The Meaning of Synthetic Gametes for Gay and Lesbian People and Bioethics Too’ (2014) 40 Journal of Medical Ethics 762.

9 Cambridge Reproduction and Progress Educational Trust, *Code of Practice for the Generation and Use of Human Stem Cell-Based Embryo Models* (2024); Emily Jackson, ‘Regulating Embryo Models in the UK’ (2024) 11 Journal of Law and the Biosciences lsae016.

10 The variety of different types of research means that there is no single right answer about what should happen in such cases, but the Medical Research Council expects researchers to adopt a policy in relation to health-related findings, and to ensure that participants are informed about this before they give consent to participation. See further Medical Research Council, *Framework on the Feedback of Health-related Findings in Research* (MRC 2014).

11 Human Tissue Authority, *Code of Practice E: Research* (HTA 2017) para 138.

12 See further, Patrick Butlin and others, ‘Consciousness in Artificial Intelligence: Insights from the Science of Consciousness’ (2023) arXiv preprint arXiv:2308; Martin Gibert and Dominic Martin, ‘In Search of the Moral Status of AI: Why Sentience is a Strong Argument’ (2022) 37 AI & Society 319.

13 Derived from the name of a mythical beast, the *Chimera*, a lion with a snake’s tail and a goat’s head, chimaeras are organisms composed of cells that are genetically distinct from each other, often because they are from different species.

14 Mototsugu Eiraku and others, ‘Self-organized Formation of Polarized Cortical Tissues from ESCs and its Active Manipulation by Extrinsic Signals’ (2008) 3 Cell Stem Cell 519.

15 Madeline A Lancaster and others, ‘Cerebral Organoids Model Human Brain Development and Microcephaly’ (2013) 501 Nature 373.

16 NAS (National Academies of Sciences, Engineering, and Medicine), *The Emerging Field of Human Neural Organoids, Transplants, and Chimeras: Science, Ethics, and Governance* (The National Academies Press 2021).

17 Gardar Arnason, Anja Pichl, and Robert Ranisch, ‘Ethical Issues in Cerebral Organoid Research’ (2023) 32 Cambridge Quarterly of Healthcare Ethics 1.

18 Jacob Jeziorski and others, ‘Brain Organoids, Consciousness, Ethics and Moral Status’ (2023) 144 Seminars in Cell & Developmental Biology 97.

19 Although validating brain organoids with an in vivo human control is in practice impossible. See further Hinterberger and Bea (n 6).

20 Hilary Putnam, *Reason*, *Truth and History* (Cambridge University Press 1981).

21 J Lomax Boyd and Nethanel Lipshitz, ‘Dimensions of Consciousness and the Moral Status of Brain Organoids’ (2024) 17 Neuroethics 5.

22 Nita A Farahany and others, ‘The Ethics of Experimenting with Human Brain Tissue’ (2018) 556 Nature 429.

23 Henry T ‘Hank’ Greely and Karola V Kreitmair, ‘Should Cerebral Organoids be Used for Research if they have the Capacity for Consciousness?’ (2021) 30 Cambridge Quarterly of Healthcare Ethics 575.

24 Cwyn Solvi, Luigi Baciadonna, and Lars Chittka, ‘Unexpected Rewards Induce Dopamine-dependent Positive Emotion–like State Changes in Bumblebees’ (2016) 353 Science 1529; Miguel Á Collado, ‘Brain Size Predicts Learning Abilities in Bees’ (2021) 19 Royal Society Open Science 201940.

25 Jonathan Birch, *The Edge of Sentience: Risk and Precaution in Humans, Other Animals, and AI* (Oxford University Press 2024).

26 Isaac H Chen and others, ‘Transplantation of Human Brain Organoids: Revisiting the Science and Ethics of Brain Chimeras’ (2019) 25 Cell Stem Cell 462.

27 Heather Browning and Walter Veit, ‘The Welfare of Brain Organoids’ (2023) 2 Molecular Psychology: Brain, Behavior, and Society 4.

28 Tim Bayne, Anil K Seth, and Marcello Massimini, ‘Are There Islands of Awareness?’ (2020) 43 Trends in Neurosciences 6.

29 Dide Jongh, Emma K Massey, and Eline M Bunnik, ‘Organoids: A Systematic Review of Ethical Issues’ (2022) 13 Stem Cell Research & Therapy 337.

30 Stefano L Giandomenico and others, ‘Cerebral Organoids at the Air–liquid Interface Generate Diverse Nerve Tracts with Functional Output’ (2019) 22 Nature Neuroscience 669.

31 Thomas Hartung and others, ‘The Baltimore Declaration Toward the Exploration of Organoid Intelligence’ (2023) 1 Frontiers in Science 1068159.

32 Lena Smirnova and others, ‘Organoid Intelligence (OI): The New Frontier in Biocomputing and Intelligence-in-a-dish’ (2023) 1 Frontiers in Science 1017235.

33 Julian Kinderlerer, ‘Organoid Intelligence: Society Must Engage in the Ethics’ (2023) Frontiers Policy Labs.

34 Insoo J Hyun, C Scharf-Deering, and Jeantine E Lunshof, ‘Ethical Issues Related to Brain Organoid Research’ (2020) 1732 Brain Research 146653. See further Cleber A Trujillo and others, ‘Complex Oscillatory Waves Emerging from Cortical Organoids Model Early Human Brain Network Development’ (2019) 25 Cell Stem Cell 558.

35 Birch (n 25).

36 Hyun and others (n 34).

37 Brett J Kagan and others, ‘In Vitro Neurons Learn and Exhibit Sentience When Embodied in a Simulated Game-world’ (2022) 110 Neuron 3952.

38 ibid.

39 Alysson R Muotri, ‘Brain Model Technology and Its Implications’ (2023) 32 Cambridge Quarterly of Healthcare Ethics 1.

40 ibid.

41 Simon Plummer and others, ‘A Human iPSC-derived 3D Platform Using Primary Brain Cancer Cells to Study Drug Development and Personalized Medicine’ (2019) 9 Scientific Reports 1407; Sara Green, Mie S Dam, and Mette N Svendsen, ‘Patient-Derived Organoids in Precision Oncology Precision oncology–Towards a Science of and for the Individual?’ in Chiara Beneduce and Marta Berolaso (eds), *Personalized Medicine in the Making: Philosophical Perspectives from Biology to Healthcare* (Springer International Publishing 2022) 125–146; Vasiliki Mollaki, ‘Ethical Challenges in Organoid Use’ (2021) 10 BioTech 12.

42 Jongh and others (n 29); Alysson R Muotri (n 39).

43 Mandeep S Singh and others, ‘Retinal Stem Cell Transplantation: Balancing Safety and Potential’ (2020) 75 Progress in Retinal and Eye Research 100779.

44 Tsutomu Sawai and others, ‘Mapping the Ethical Issues of Brain Organoid Research and Application’ (2022) 13 AJOB Neuroscience 81.

45 Masanori Kataoka and others, ‘Are Human Brain Organoids Cloned Human Individuals? An Ethical Analysis’ (2023) 2 Molecular Psychology: Brain, Behavior, and Society 18.

46 Farahany and others (n 22).

47 Bryan Jennett and others, ‘Transplants: Are The Donors Really Dead?’ (1980) 281 British Medical Journal 1139. Described as ‘a farrago of inaccuracy’, it took 10 years for organ donation rates to be restored after the airing of Panorama’s *Are Transplant Donors Really Dead?.* See further Carl Gray, ‘Twice Dead: Organ Transplants and the Reinvention of Death’ (2002) 324 British Medical Journal 1401.

48 Lulu Ding and others, ‘Knowledge Graphs of Ethical Concerns of Cerebral Organoids’ (2022) 55 Cell Proliferation e13239; Takuya Niikawa and others, ‘Human Brain Organoids and Consciousness’ (2022) 15 Neuroethics 5.

49 Andrea Nani, Gabriele Volpara, and Andrea Faggio, ‘Sentience With or Without Consciousness’ (2021) 28 Journal of Consciousness Studies 60.

50 Alasdair Cochrane, *Sentientist Politics: A Theory of Global Inter-Species Justice* (Oxford University Press 2018). See also Jeff McMahan, ‘Suffering and Moral Status’ in Steve Clarke, Hazem Zohny, and Julian Savulescu (eds), *Rethinking Moral Status* (Oxford Academic 2021).

51 Greely and Kreitmair (n 23).

52 Arun Sharma, Peter Zuk, and Christopher Thomas Scott, ‘Scientific and Ethical Uncertainties in Brain Organoid Research’ (2021) 21 The American Journal of Bioethics 48.

53 Arnason and others (n 17).

54 Sarah Diner, ‘Potential Consciousness of Human Cerebral Organoids: on Similarity-based Views in Precautionary Discourse’ (2023) 16 Neuroethics 23.

55 Jeziorski and others (n 18).

56 Heather Browning and Walter Veit, ‘The Sentience Shift in Animal Research’ (2022) 28 The New Bioethics 299.

57 Rachel A Ankeny and Ernst Wolvetang, ‘Testing the Correlates of Consciousness in Brain Organoids: How Do We Know and What Do We Do?’ (2021) 21 The American Journal of Bioethics 51.

58 Farahany and others (n 22).

59 Ankeny and Wolvetang (n 57).

60 Julian Koplin, Olivia Carter, and Julian Savulescu, ‘Moral Status of Brain Organoids’ in Steve Clarke, Hazem Zohny, and Julian Savulescu (eds), *Rethinking Moral Status* (Oxford Academic 2021), citing Elan L Ohayon, Paul W Tsang, and Ann Lam, ‘A Computational Window into the Problem With Organoids: Approaching Minimal Substrates for Consciousness’ *Paper presented at the Neuroscience 2019 Conference* (Chicago).

61 Masanori Kataoka and others, ‘The Ethics of Human Brain Organoid Transplantation in Animals’ (2023) 16 Neuroethics 27.

62 Koplin and Savulescu (n 3); Jonathan Birch and Heather Browning, ‘Neural Organoids and the Precautionary Principle’ (2021) 21 The American Journal of Bioethics 56.

63 Thomas Nagel, ‘What Is It Like to Be a Bat?’ (1974) 83 The Philosophical Review 435.

64 Smirnova and others (n 32).

65 Birch and Browning (n 62).

66 Julian J Koplin, Christopher Gyngell, and Julian Savulescu, ‘Germline Gene Editing and the Precautionary Principle’ (2020) 34 Bioethics 49.

67
*Rio Declaration on Environment and Development* (United Nations 1992).

68 Stephen John, ‘Risk and Precaution’ in A Dawson (ed), *Public Health Ethics: Key Concepts and Issues in Policy and Practice* (Cambridge University Press 2011) 67–84.

69 Jonathan Birch, ‘Animal Sentience and the Precautionary Principle’ (2017) 2 Animal Sentience 1.

70 Jeziorski and others (n 18).

71 Niikawa and others (n 48).

72 Andrea Lavazza and Marcello Massimini, ‘Cerebral Organoids: Ethical Issues and Consciousness Assessment’ (2018) 44 Journal of Medical Ethics 606. See also Henri-Corto Stoeklé, Geneviève Marignac, and Christian Hervé, ‘Macro-bio-ethical Versus Micro-bio-ethical Issues Concerning Human Brain Organoids’ (2023) 14 AJOB Neuroscience 199.

73 Tomasz Żuradzki, ‘Against the Precautionary Approach to Moral Status: The Case of Surrogates for Living Human Brains’ (2021) 21 The American Journal of Bioethics 53.

74 ibid.

75 Denise Meyerson, ‘Is there a Right to Access Innovative Surgery?’ (2015) 29 Bioethics 342.

76 William MS Russell and Rex L Burch, *The Principles of Humane Experimental Technique* (Methuen & Co 1959).

77 Tom Beauchamp and David DeGrazia, *Principles of Animal Research Ethics* (Oxford University Press 2019).

78 Koplin and Savulescu (n 3).

79 ibid.

80 Katherine Bassil and Dorothee Horstkötter, ‘Ethical Implications in Making Use of Human Cerebral Organoids for Investigating Stress—Related Mechanisms and Disorders’ (2023) 32 Cambridge Quarterly of Healthcare Ethics 1.

81 Henry T Greely, ‘Human Brain Surrogates Research: The Onrushing Ethical Dilemma’ (2021) 21 The American Journal of Bioethics 34.

82 Andrew J Barnhart and Kris Dierickx, ‘A RAD Approach to iBlastoids with a Moral Principle of Complexity’ (2022) 22 The American Journal of Bioethics 2254.

83 J Lomax Boyd and Nethanel Lipshitz (n 25).

84 Oscar Horta, ‘Why the Concept of Moral Status Should be Abandoned’ (2017) 20 Ethical Theory and Moral Practice 899; Benjamin Sachs, ‘The Status of Moral Status’ (2011) 92 Pacific Philosophical Quarterly 87.

85 Cochrane (n 50).

86 Jeremy Bentham, *Introduction to Principles of Morals and Legislation* [1789] (Clarendon Press 1879).

87 Peter Singer, *Animal Liberation* (Harper Collins 2009).

88 Philosophy for Our Times Podcast, *On Humans and Animals: Peter Singer and Mary Midgley* (2024) <https://player.fm/series/philosophy-for-our-times/on-humans-and-animals-peter-singer-mary-midgley> accessed 15 December 2024.

89 McMahan (n 50).

90 James Rachels, *Created from Animals: The Moral Implications of Darwinism* (Oxford University Press 1991). For a different view, see Cochrane (n 50).

91 Martha C Nussbaum, ‘The Moral Status of Animals’ (2006) 52 Chronicle of Higher Education B6–8.

92 Jessica Pierce, ‘The Dying Animal’ (2013) 10 Journal of Bioethical Inquiry 469.

93 Julian J Koplin and Christopher Gyngell, ‘Emerging Moral Status Issues’ (2020) 38 Monash Bioethics Review 95.

94 J Lomax Boyd and Nethanel Lipshitz (n 25).

95 Steve Clarke and Julian Savulescu, ‘Rethinking our Assumptions about Moral Status’ in Steve Clarke, Hazem Zohny, and Julian Savulescu (eds), *Rethinking Moral Status* (Oxford Academic 2021).

96 Joshua Shepherd, ‘The Moral Status of Conscious Subjects’ in Steve Clarke, Hazem Zohny, and Julian Savulescu (eds), Rethinking Moral Status (Oxford Academic 2021).

97 Udo Schuklenk, ‘Moral Recognition and the Limits of Impartialist Ethics: On Androids, Sentience, and Personhood’ in Steve Clarke, Hazem Zohny, and Julian Savulescu (eds), *Rethinking Moral Status* (Oxford Academic 2021).

98 Bjørn Hofmann, ‘Making and Managing New Biological Entities: Conceptual, Ontological, Epistemological, and Ethical Aspects’ (2023) 66 Perspectives in Biology and Medicine 211; Mollaki (n 41); Sarah N Boers and others, ‘Organoids as Hybrids: Ethical Implications for the Exchange of Human Tissues’ (2019) 45 Journal of Medical Ethics 131.

99 Hinterberger and Bea (n 6). See also Thomas Hartung, Itzy E Morales Pantoja, and Lena Smirnova, ‘Brain Organoids and Organoid Intelligence from Ethical, Legal, and Social Points of View’ (2024) 6 *Frontiers in Artificial Intelligence* 1307613.

100 Bristol Royal Infirmary Inquiry, *Interim Report: Removal and Retention of Human Material* (May 2000); *Report of the Royal Liverpool Children’s (Alder Hey) Inquiry*, House of Commons (January 2001) (Redfern Report).

101 Masanori Kataoka and others, ‘The Donation of Human Biological Material for Brain Organoid Research: The Problems of Consciousness and Consent’ (2024) 30 Science and Engineering Ethics 1.

102 Juli Bollinger and others, ‘Patients’ Perspectives on the Derivation and Use of Organoids’ (2021) 16 Stem Cell Reports 1874.

103 Boers and others (n 98).

104 Henry T Greely, ‘The Dilemma of Human Brain Surrogates: Scientific Opportunities, Ethical Concerns’ in A D’Aloia and MC Errigo (eds), *Neuroscience and Law: Complicated Crossings and New Perspectives* (Springer 2020) 371–399. Masanori Kataoka and others (n 101).

105 Sietske AL van Till and Eline M Bunnik, ‘Symbolic Value of Brain Organoids: Shifting the Focus from Consciousness to Sociocultural Perspectives on Resemblance’ (2023) 14 AJOB Neuroscience 210.

106 See further Imogen Goold, Kate Greasley, Jonathan Herring, and Loane Skene (eds), *Persons, Parts and Property: How Should we Regulate Human Tissue in the 21st Century?* (Bloomsbury Publishing 2014).

107 In *Yearworth v North Bristol NHS Trust*, [2009] EWCA Civ 37, men whose sperm samples had been damaged as a result of the defendant Trust’s negligence were said to have ownership rights over their sperm in relation to the Trust.

108 Jesse Wall, ‘The Legal Status of Body Parts: A Framework’ (2011) 31 Oxford Journal of Legal Studies 783.

109 Lili Zhu and others, ‘Patent Bibliometric Analysis for Global Trend of Organoid Technologies in the Past Decade’ (2022) 25 IScience 104728.

110 European Patent Convention, art 53(a).

111 If, like Henrietta Lacks’ biopsy in 1951, one person’s cells had particular and long-lasting value in organoid technology, then, as with the Henrietta Lacks case, they or their family might seek to claim that there should be some benefit-sharing of the profits, which derived from their cultured cells. See further Rebecca Skloot, *The Immortal Life of Henrietta Lacks* (Broadway Paperbacks 2017).

112 Hinterberger and Bea (n 6).

113 NAS (n 16).

114 Abed AlFatah Mansour and others, ‘An In vivo Model of Functional and Vascularized Human Brain Organoids’ (2018) 36 Nature Biotechnology 432.

115 H Isaac Chen and others, ‘Transplantation of Human Brain Organoids: Revisiting the Science and Ethics of Brain Chimeras’ (2019) 25 Cell Stem Cell 462.

116 ibid.

117 Farahany and others (n 22).

118 HI Chen and others, ‘Transplantation of Human Brain Organoids: Revisiting the Science and Ethics of Brain Chimeras’ (2019) 25 Cell Stem Cell 462.

119 ibid; Jongh and others (n 29).

120 T Kitahara and others, ‘Axonal Extensions Along Corticospinal Tracts from Transplanted Human Cerebral Organoids’ (2020) 15 Stem Cell Reports 467.

121 NAS (n 16).

122 ibid.

123 ibid.

124 Jason Scott Robert and Françoise Baylis, ‘Crossing Species Boundaries’ (2003) 3 American Journal of Bioethics 1.

125 ibid.

126 Philip Karpowicz, Cynthia Cohen, and Derek van der Kooy, ‘Developing Human-nonhuman Chimeras in Human Stem Cell Research: Ethical Issues and Boundaries’ (2005) 15 Kennedy Institute of Ethics Journal 107.

127 Benjamin Capps, ‘What do Chimeras Think About?’ (2023) 32 Cambridge Quarterly of Healthcare Ethics 96.

128 Takahiro Kitahara and others, ‘Axonal Extensions Along Corticospinal Tracts from Transplanted Human Cerebral Organoids’ (2020) 15 Stem Cell Reports 467.

129 Hyun and others (n 34).

130 Julian Koplin and Dominic Wilkinson, ‘Moral Uncertainty and the Farming of Human-pig Chimeras’ (2019) 45 Journal of Medical Ethics 440.

131 Mollaki (n 41).

132 Jongh and others (n 29).

133 Hinterberger and Bea (n 6).

134 Ibid.

135 Koplin and Savulescu (n 3).

136 Greely and Kreitmair (n 23).

137 Mary Warnock, ‘Moral Thinking and Government Policy: The Warnock Committee on Human Embryology’ (1985) 63 Milbank Memorial Fund Quarterly: Health and Society 504.

138 Human Tissue Act 2004 Schedule 1 para 5.

139 Human Tissue Act 2004 s 1(1).

140 Human Tissue Authority (n 11) para 39.

141 Human Tissue Act 2004, s 3(2)(6)(ba).

142 ibid s 3(2).

143 Mental Capacity Act 2005, s 1.

144 Medical Research Council, *Research and the Human Tissue Act 2004: Consent* (MRC 2019).

145 HTA (n 11) para 63.

146 Human Tissue Authority (n 11) para 10.

147 ibid para 11.

148 Recognized RECs are ethics committees recognized by the UK Ethics Committee Authority under the Clinical Trials Regulations. In practice, this means NHS RECs.

149 Human Tissue (Quality and Safety for Human Application) Regulations 2007 para 120(b).

150 UKRI, *Code of Practice for the use of Human Stem Cell Lines* (UKRI 2010).

151 <https://ebisc.org/docs/ebisc/EBiSC_2_EAUA.pdf> accessed 4 September 2024.

152 International Society for Stem Cell Research, *Guidelines for Stem Cell Research and Clinical Translation* (ISSCR 2021), recommendation 2.2.

153 ibid para 2.2.1A.

154 Julian Koplin and John Massie, ‘Lessons from Frankenstein 200 Years on: Brain Organoids, Chimaeras and Other “Monsters”’ (2021) 47 Journal of Medical Ethics 567.

155 ibid.

156 International Society for Stem Cell Research (n 152).

157 Insoo Hyun and others, ‘ISSCR Guidelines for the Transfer of Human Pluripotent Stem Cells and Their Direct Derivatives into Animal Hosts’ (2021) 16 Stem Cell Reports 1409.

158 ibid.

159 ibid.

160 ibid.

161 David DeGrazia, *Taking Animals Seriously: Mental Life and Moral Status* (Cambridge UP 1996).

162 Julian J Koplin, ‘Response to the ISSCR Guidelines on Human–animal Chimera Research’ (2022) 37 Bioethics 192.

163 ibid.

164 It is commonly assumed that embryos are not sentient until around 20 weeks, but there have been suggestions that a lower threshold, perhaps of around 12 weeks, would represent an appropriately precautionary approach to fetal sentience. See further, Stuart W Derbyshire and John C Bockmann, ‘Reconsidering Fetal Pain’ (2020) 47 Journal of Medical Ethics 3; Anna Ciaunica, Adam Safron, and Jonathan Delafield-Butt, ‘Back to Square One: The Bodily Roots of Conscious Experiences in Early Life’ (2021) 2 Neuroscience of Consciousness niab037.

165 Department of Health and Social Security, *Report of the Committee of Enquiry into Human Fertilisation and Embryology* (HMSO 1984) (Warnock Report) para 11.15.

166 Hansard 5 December 2002, col 1327.

167 John Robertson, ‘Symbolic Issues in Embryo Research’ (1995) 25 Hastings Center Report 37.

168 Lisa Bortolotti and John Harris, ‘Embryos and Eagles: Symbolic Value in Research and Reproduction’ (2006) 15 Cambridge Quarterly of Healthcare Ethics 22.

169 Human Fertilisation and Embryology Act 1990, Schedule 2(3A).

170 Schedule 2(3)(5).

171 Human Fertilisation and Embryology Act 1990 Schedule 3(2)(c).

172 Warnock Report (n 165) para 11.5, defined the primitive streak as: ‘a heaping-up of cells at one end of the embryonic disc on the fourteenth or fifteenth day after fertilisation’. Two primitive streaks may form in a single embryonic disc. This is the latest stage at which identical twins can occur. The primitive streak is the first of several identifiable features which develop in and from the embryonic disc during the succeeding days, a period of very rapid change in the embryonic configuration. By the seventeenth day the neural groove appears and by the twenty-second to twenty-third day this has developed to become the neural folds, which in turn start to fuse and form the recognizable antecedent of the spinal cord’.

173 Human Fertilisation and Embryology Act 1990, s 3.

174 Audrey R Chapman, ‘Brain Models in a Dish: Ethical Issues in Developing Brain Organoids’ (2019) 10 AJOB Neuroscience 113.

175 Bernard E Rollin, ‘The Regulation of Animal Research and the Emergence of Animal Ethics: A Conceptual History’ (2006) 27 Theoretical Medicine and Bioethics 285.

176 s 5B(3)(b).

177 s 5(B)(3)(d).

178 Tine Ravn and others, ‘Public Perceptions and Expectations: Disentangling the Hope and Hype of Organoid Research’ (2023) 18 Stem Cell Reports 841; Sarah Franklin and Emily Jackson, *The 14 Day Rule and Human Embryo Research: a Sociology of Biological Translation* (Routledge 2024); van Till and Bunnik (n 105).

179 Ravn and others ibid; HYBRIDA: Regulating Organoid Research: Embedding a Comprehensive Ethical Dimension to Organoid-based Research and Related Technologies, D4.3 *Public Attitudes, Understandings and Perspectives on Organoid Research: Findings from a Series of Deliberative Workshops* (2022) <https://pure.au.dk/portal/en/publications/d43-public-attitudes-understandings-and-perspectives-on-organoid-> accessed 15 December 2024.

180 Ravn and others ibid; Dolly R Haselager and others, ‘Breeding Brains? Patients’ and Laymen’s Perspectives on Cerebral Organoids’ (2020) 15 Regenerative Medicine 2351.

181 Bollinger and others (n 102).

182 Ravn and others (n 178).

183 Bollinger and others (n 102).

184 Sietske AL van Till and others, ‘An Assessment of the Moral Value of Neuronal Cell Models and Brain Organoids’ (2023) 2 Molecular Psychology: Brain, Behavior, and Society 15.

185 Peter B Reiner, ‘The Rise of Neuroessentialism’ in Judy Illes and Barbara J Sahakian (eds), *The Oxford Handbook of Neuroethics* (Oxford University Press 2012) ch 10.

186 Kataoka and others (n 45).

187 Philip Ball, *How to Grow a Human: Adventures in How We Are Made and Who We Are* (Chicago University Press 2019).

188 Koplin and others (n 60) (emphasis in original).

189 Ball (n 187).

190 Masanori Kataoka, Tsung-Ling Lee, and Tsutomu Sawai, ‘The Legal Personhood of Human Brain Organoids’ (2023) 10 Journal of Law and the Biosciences lsad007.

191 Andrew J Barnhart and Kris Dierickx, ‘Cultures and Cures: Neurodiversity and Brain Organoids’ (2021) 22 BMC Medical Ethics 1–6.

192 ibid.

193 Jeremy Sugarman and others, ‘Critical Considerations for Public Engagement in Stem Cell-related Research’ (2023) 18 Stem Cell Reports 420.

194 Cathelijne M Reincke, Annelien L Bredenoord, and Marc HW van Mil, ‘From Deficit to Dialogue in Science Communication: the Dialogue Communication Model Requires Additional Roles from Scientists’ (2020) 21 EMBO Reports e51278.

195 Ana S Iltis and others, ‘Ethical, Legal, Regulatory, and Policy Issues Concerning Embryoids: A Systematic Review of the Literature’ (2023) 14 Stem Cell Research & Therapy 1; Bernard Baertschi and others, ‘Organoids Research: What are the Ethical Issues?’ (2020) HAL Open Science ffinserm-03117706.

196 Bollinger and others (n 102).

197 Iltis and others (n 195).

198 Abigail Presley, Leigh Ann Samsa, and Veljko Dubljević, ‘Media Portrayal of Ethical and Social Issues in Brain Organoid Research’ (2022) 17 Philosophy, Ethics, and Humanities in Medicine 1.

199 Felix Gille and Caroline Brall, ‘Can We Know if Donor Trust Expires? About Trust Relationships and Time in the Context of Open Consent for Future Data Use’ (2022) 48 Journal of Medical Ethics 184.

200 Ipsos MORI, *Consent to Use Human Tissue and Linked Health Data in Health Research: A Public Dialogue for Health Research Authority and Human Tissue Authority* (Ipsos MORI 2018).

201 Cambridge Reproduction and Progress Educational Trust (n 9).

202 Ana M Pereira Daoud and others, ‘Modelling Human Embryogenesis: Embryo-like Structures Spark Ethical and Policy Debate’ (2020) 26 Human Reproduction Update 779.

203 Birch (n 25).

204 Rollin (n 175).

205 Home Office, *Experiments on Living Animals: Return of the Experiments Performed under the Cruelty to Animals Act 1876, during 1972* (HMSO 1973).

206
*Statistics of Scientific Procedures on Living Animals: Great Britain 2022* (Home Office 2023).

207 Kataoka and others (n 190).

208 Farahany and others (n 22).

209 Nuffield Council on Bioethics, *Briefing Note: Neural Organoids in Research: Ethical Considerations* (NCOB 2024).

210 Nuffield Council on Bioethics, *Call for Evidence: Neural Organoids (Decision-making and Governance)* (NCOB 2024).

211 Joshua Jowitt, ‘Agency, Moral Worth and the Legal Status of Human Cerebral Organoids’ (2023) 2 Molecular Psychology: Brain, Behavior, and Society 12.

212 ibid.

213 Warnock Report (n 165).

